# Editorial: Expanding spectrum of primary aldosteronism: exploring new grounds

**DOI:** 10.3389/fendo.2023.1171857

**Published:** 2023-04-18

**Authors:** Norlela Sukor, Troy H. Puar, Sarat Sunthornyothin, Nor Azmi Kamaruddin

**Affiliations:** ^1^ Department of Medicine, Faculty of Medicine, Universiti Kebangsaan Malaysia, Kuala Lumpur, Malaysia; ^2^ Hospital Canselor Tuanku Muhriz, Jalan Yaacob Latif, Bandar Tun Razak, Kuala Lumpur, Malaysia; ^3^ Department of Endocrinology, Changi General Hospital, Singapore, Singapore; ^4^ Department of Medicine, Faculty of Medicine, Chulalongkorn University, Bangkok, Thailand; ^5^ Department of Cardiology, National Heart Institute, Kuala Lumpur, Malaysia

**Keywords:** primary aldosteronism, aldosterone producing adenoma, adrenalectomy, Mineralocorticoid receptor (MR) antagonist, cardiovascular, adrenal venous sampling, stroke, obstructive sleep apnea

Seven decades have lapsed since the first description of primary aldosteronism (PA) by Jerome W. Conn (1). Despite a significant time-lapse, PA remains an underrated cause of hypertension with devastating cardiovascular and renal complications (2, 3).

This Research Topic gathers different contributions highlighting the latest advances in the diagnosis and management of PA. In the first article by Parasiliti-Caprino et al., the accuracy of simple and adjusted aldosterone indices for assessing selectivity and lateralization of adrenal vein sampling (AVS) is evaluated. AVS is regarded as the gold standard test for PA subtyping (4). However, AVS is technically difficult and failure to cannulate both adrenal veins render the test inconclusive. The authors demonstrated the utility of aldosterone indices to determine both selectivity and lateralization. These indices could potentially be useful when only one adrenal vein is successfully cannulated, and their utility should be determined in future prospective trials.

The second article highlights the importance of accurate catheter tip placement during AVS which is critical in the interpretation of the results. Traditionally, the ratio of plasma cortisol concentration in the adrenal vein to peripheral vein defines the selectivity index (SI). However, with no standardized cut-off levels, this limits the applicability of AVS when cortisol is being utilized (5, 6). The use of free metanephrine (FMN) has been shown to be superior to cortisol in assessing SI (7, 8). This study shows that FMN is a better analyte than cortisol in confirming the correct placement of the catheter’s tip. The main limitation, however, was that no confirmatory tests were performed. Other studies have also explored the use of different analytes such as aldosterone-to-renin ratio (ARR) in combination with cortisol SI (9) and plasma metanephrines in addition to cortisol (10). While the results are promising, further research is needed to establish the utility of these markers especially in those with confirmed PA.

The diagnosis of PA requires the demonstration of an unsuppressed aldosterone level by various suppression tests (11). The oral salt loading test (OSLT) has been widely used in the diagnostic work-up, albeit its accuracy has been challenged in recent years. In the third article, Ozeki et al. present a new chemiluminescent enzyme immunoassay (CLEIA) based on a two- step sandwich method to measure 24-hour urine aldosterone excretion. The accuracy of aldosterone measurement in urine samples using various methods have also been examined previously. Liquid chromatography-tandem mass spectrometry (LC-MS/MS) was noted to have the highest accuracy (12). The lower cut-off value suggested for PA diagnosis needs to be validated in a larger clinical trial.

The fourth article focuses on the role of urinary extracellular vesicles (uEVs) sodium chloride cotransporter (NCC) in PA subtyping. In view of the invasive nature and technical difficulties of AVS, identifying a non-invasive alternative is crucial. The authors show that NCC can be potentially used to distinguish between different subtypes of PA as the expression of phosphorylated form of NCC (pNCC) in uEVs are different in various subtypes and genotypes. Previous studies have explored the use of uEVs as a biomarker in lupus nephritis and diabetic nephropathy where the presence of uEVs provide insights into the pathophysiology and aid the diagnosis and management (13, 14). The main limitations of this study include the small sample size and the lack of a validation cohort. Although the study highlights the potential of using uEVs as a non-invasive biomarker to subtype PA, further research in this field is warranted.

In the fifth article, Nguyen et al. recruited 300 patients with a recent stroke and reported the prevalence of PA amongst those with hypertension, resistant hypertension and hypertension with atrial fibrillation (AF) as 4%, 11% and 30% respectively. Previous studies have shown that patients with PA are at increased risk of stroke and AF (15, 16). This study highlights the importance of screening for PA, particularly in high-risk groups of patients. This approach will enable early detection and treatment of a potentially reversible cause of hypertension and stroke.

The sixth article by Puar et al. demonstrated that PA treatment improved subclinical left ventricular (LV) systolic function, using speckle-tracking echocardiography to assess LV global longitudinal strain (GLS). While previous studies have shown that patients with PA have impaired LV systolic function (17), this study demonstrated that surgery led to better improvement compared to medical therapy, and that reversal of renin suppression may be a key factor in improving systolic function.

The seventh article by Huang et al. investigates the relationship between vascular ageing and left ventricular concentric geometry (LVCG) in patients with newly diagnosed PA. In this study carotid intima-media thickness (cIMT) is significantly associated with left ventricular hypertrophy (LVH) whilst brachial-ankle pulse wave velocity (baPWV) is significantly associated with LVCG. While the study has some limitations, such as its retrospective and single-center design, its findings are consistent with previous research. Wang et al. (18) demonstrate a positive correlation between baPWV and left ventricular mass index in hypertensive patients. This study provides important insights into the early assessment of cardiac damage in newly diagnosed PA.

The review article by Ahmed and Hundemer provides a comprehensive literature search encompassing studies from the past two decades supporting surgical adrenalectomy as the preferred treatment option for unilateral PA compared to medical therapy. Adrenalectomy is highly successful in reversing the clinical and biochemical abnormalities, mitigating long-term risks, and offering the potential for disease cure. Several other studies also favored surgical approach with significant reductions in blood pressure, glucose, number of medications used, and cardiovascular events and mortality (2, 3, 19). This review underscores the importance of personalized treatment plans for improved patient outcomes.

Tetti et al. in their review provide an extensive overview of the current knowledge on the molecular and cellular mechanisms that contribute to the pathogenesis of PA. The authors focus on recent advances in the understanding of the disrupted cell growth mechanisms in PA through the combined application of transcriptomics, metabolomics, and epigenetics. The review is a valuable resource for clinicians as it highlights the key findings in the field, fill up the gaps in the literature and explore areas where further research is needed.

Loh and Sukor presented a review article that collates and puts into perspective current available research on the association between PA and obstructive sleep apnea (OSA). Given the high prevalence of hypertension, PA, and OSA, understanding their potential association and clinical implications is important. The authors critically analyzed the existing literature and identified several limitations in the currently available studies, which include heterogeneity in the study designs and populations, lack of uniform criteria for diagnosis and treatment of PA and OSA, and potential confounding factors such as obesity and diabetes. Although other studies have also suggested a potential link between PA and OSA (20, 21), there is a need for further research to elucidate the relationship between these two conditions.

A large majority of operated aldosterone-producing adenomas (APAs) harbour known somatic mutations, which are associated with membrane depolarisation and increased aldosterone production. However, mechanisms driving cell proliferation of these adenomas remained unknown. Abdellatif et al. summarizes the current knowledge on known regulators of adrenal growth and function. In addition, they have focused on the interplay between the hormonal and vascular interfaces which may explain the development of APAs and PA. The strength of this review lies in its comprehensive coverage of the current knowledge on the topic, drawing from various research disciplines including endocrinology, molecular genetics and vascular biology.

In conclusion, this Research Topic provides a timely overview of the latest advances in the diagnosis and treatment of PA and its associated sequelae. The articles included in this Research Topic provide valuable insights into the expanding pathophysiology of PA and the need for ongoing research to fill up the gap of this important disease entity.

## Author contributions

All authors listed have made a substantial, direct, and intellectual contribution to the work and approved it for publication.
